# Eye-Glasses and Spectacles—The Wearing of and Care of Them

**Published:** 1892-12

**Authors:** Wm. H. Baldinger

**Affiliations:** Galveston, Texas


For Daniel's Texas Medical Journal.

### EYE-GLASSES AND SPECTACLES—THE WEARING OF AND CARE OF THEM.

BY WM. H. BALDINGER, M. D., GALVESTON, TEXAS.

TO EVERY person who, by nature, accident or old age, is compelled to aid vision by use of glasses, the important questions often arise:

1. Who should, or should not wear glasses?
2. If a necessity, what kind to wear?
3. Who should be consulted in order to determine the need of glasses?
4. The care of glasses.

In this age of close confinement, constant and exacting near work, eye-strain is much more prevalent than even a decade ago. The oculist, who by his scientific knowledge and skill in determining accurately all errors of refraction, imbalance of the muscles of the eyeballs and knowledge of the various diseases of the eye, many of which produce obscure vision, is, beyond all doubt, the proper person to consult.

The oculist, by his experience and training in large clinics and observation in private practice, decides the question as to whether the applicant will be benefited by wearing glasses, and furnishes or prescribes the formula for the guidance of the optician, who, by his qualifications and skilled training in the workshop produces the perfectly ground lens, comfortable and accurately fitting frames.

The spheres of the oculist and optician are different in degree, the former including the scientific, the latter the artistic, the one dependent on the other in carrying to a successful end the full benefits to be derived from the wearing of properly adjusted and fitted glasses. The sooner the public is educated to the several spheres of the oculist and optician, viz: the oculist being the proper person to consult and determine whether or not the wearing of glasses is necessary, the optician the one to grind the lenses and fit the frames according to the oculist's formula or prescription, the more will be the benefit bestowed on suffering humanity, and the appreciation of the great blessing and comfort of properly adjusted and fitted glasses.

Obscuration of vision being due either to disease of the eye or appendages, or errors of refraction, it is reasonable to conclude that all such cases or conditions are not improved by simply wearing lenses or glasses.

Errors of refraction, whether acquired or the result of the cropping out, due to muscular fatigue, the patient being unconscious of the existing error until made cognizant by the refusal of the muscle or muscles to longer carry the burden. This is the usual course of slight degrees of the errors known as astigmatism, simple, compound, or mixed.

Hyperopia (or far sight) is a potent factor next in importance, whilst myopia (or near sight) is the least, owing to inconvenience of short range and narrow limits of vision and field of observation.

Myopia or hyperopia combined with astigmatism, is apt to produce a train of symptoms of harrowing pains and bewildering aches of neck and head.

With properly adjusted lenses of correct refracting media, exact fitting frames and constant wearing of spectacles, the painful eyeballs, aching brow or severe, constant headaches, due to errors of refraction, disappear, the patient once more assuming his (or her) duties of life, diligently and with much pleasure, which had been hitherto a source of annoyance and discomfort. By the correction of the errors of refraction, to the myope (or near sighted) a new world is discovered; to the hyperope (or far sighted) a comfort and alleviation of painful aches inaugurated; to the myope or hyperope additionally affiicted with astigmatism, double relief is given, by dispelling the many vague pains and aches attendant on these errors of refraction.

Thus the wearing of spectacles or eye-glasses of proper focal strength, will overcome many a headache, in the hyperope or astigmatic-hyperope and astigmatic-myope, and heartache in the sensitive myope, who per nature of short range of vision is daily accused by companions or acquaintances for slights of recognition (unintentional) on street, at theatre or public reception.

It need only be mentioned that the blessings of improved vision is appreciated alone by those having regained vision with the aid of scientifically adjusted and skillfully fitted lenses.

For children, the spectacle with hook frame recommends itself, for economic reasons. For the constant wearer, with astigmatic error, by reason of firm adaptation and stationary position of of lens, the spectacle is alone recommended, since by wearing *pince-nez* (eye-glasses) the axis of the cylinder is apt to malposition, thus defeating the objects of the correction.

To those individuals having a nose adapted to the wearing of eye-glasses, and having need of the assistance of lenses occasionally for near work or distance, the eye-glass is recommended.

Whether the frames are to be wrought of gold, steel, or other metal, must be determined by the wearer, the strength, lightness of weight and durability, should be considered paramount. The optician attending, by his experience and training, to the proper grinding of lens, comfortably fitting frames and other necessary details.

To derive the most benefit from glasses prescribed, the lenses (or glasses) should always be kept scrupulously clean (with chamois or soft rag to prevent scratching), be well centered, and at a proper distance from the eyes.

By noting these seemingly trivial points, the aid of glasses will prove of great advantage and untold blessings to those handicapped by errors of refraction.

